# Statins in Patients with Chronic Kidney Disease: Evidence from Systematic Reviews and Randomized Clinical Trials

**DOI:** 10.1371/journal.pmed.0030123

**Published:** 2006-05-30

**Authors:** Sankar D Navaneethan, Francesca Pansini, Giovanni F. M Strippoli

## Abstract

Good evidence supports the widespread use of statins for cardiovascular disease; there is less evidence for their use in kidney disease. The question is addressed by discussing published trials and systematic reviews.

Statins are recommended for primary and secondary prevention of cardiovascular disease in the general population with elevated low-density lipoprotein (LDL) cholesterol based on extensive randomized trial evidence of cardiovascular benefits. Statin use in patients with kidney disease, including patients on dialysis, is also increasing, although fewer and smaller trials exist. Is the use of statins in patients with chronic kidney disease supported by the available randomized clinical trial evidence? In this article, the question was addressed by reviewing current published trials or systematic reviews on this topic. Available data confirm the cardiovascular benefits of statins in patients with chronic kidney disease, although their impact on all-cause mortality is still not established. Statins are safe in these high-risk patients.

## Statins in Patients with Kidney Disease: Evidence from Systematic Reviews and Randomized Clinical Trials

### Case scenario

A 72-year-old, diabetic white male on dialysis for six months presents for a routine office visit. He has a total cholesterol level of 190 mg/dl, LDL cholesterol level of 136 mg/dl, high-density lipoprotein (HDL) cholesterol level of 37 mg/dl, and triglycerides level of 192 mg/ dl. Given his dialysis status, clinical history of diabetes, and presence of hyperlipidemia, this is a patient at high risk for cardiovascular events. He is very concerned about the high cholesterol levels, and you discuss the option of starting treatment with a lipid-lowering agent (statins). However, before making that decision, you reschedule him for another visit within one week and propose that you will have a look at the most updated literature—particularly meta-analysis and randomized clinical trials data, with a major focus on patients on dialysis—having recently heard that a new trial conducted in diabetic patients on dialysis found no significant improvements in cardiac end points with statins. You are also aware of the significant toxicity associated with statins and, hence, would like to make the best “evidence-based” decision for your patient, based on a benefit–harm trade-off.

### Background

Cardiovascular diseases account for approximately 50% of deaths in patients with end-stage renal disease [ 1]. Patients on dialysis with concomitant cardiovascular disease have higher all-cause mortality rates compared with the general population. The prevalence of traditional cardiovascular risk factors such as diabetes and hypertension is also higher in patients on dialysis compared with the general population. Dyslipidemia is common in this group, with mildly elevated LDL cholesterol levels and a marked predominance of highly atherogenic, small, dense LDL particles [ 2]. Paradoxically, but not surprisingly given their biased nature when assessing intervention issues, some observational studies have found a significant increase while others showed a significant decrease in the risk of death in individuals with lower cholesterol levels and chronic kidney disease [ 3–6]. Additionally, currently available randomized clinical trials in chronic kidney diseases are few and small, and often provide data on surrogate end points alone, with limited information on mortality and cardiovascular event rates.

Despite conflicting results and a scant evidence base, statins have been recommended broadly in patients on dialysis by various guidelines, on the grounds of beneficial effects found in observational data or in trials conducted both in populations on dialysis and in other populations (those not on dialysis). The National Kidney Foundation–Kidney Disease Outcomes Quality Initiative guidelines recommend the use of statins in chronic kidney disease stages 3, 4, and 5 (stage 3 glomerular filtration rate [GFR], 30–59 ml/min; stage 4 GFR, 15–29 ml/min; stage 5 GFR, less than 15 ml/min) with LDL levels greater than 130 mg/dl [ 7]. Globally, more than 1.3 million people receive dialysis, and more than 600,000 patients receive dialysis in the United States and Europe alone [ 8]. Statin use in patients on dialysis varies according to the geographic region, with at least 40%–50% of US patients on dialysis receiving statin therapy [ 7]. Given the paucity of evidence in this field, there appears to be conflict between recommendations from guidelines, evidence, and current practice patterns. Furthermore, given the significantly larger number of patients taking statins and being cared for by internists, it is imperative to review the evidence supporting its use.

## Systematic Reviews of Statins in Patients with Chronic Kidney Disease

You performed a literature search in the Renal Health Library ( http://www.cochrane-renal.org/renalhealthlibrary.php), which is produced by the Cochrane Renal Group, and includes the most updated list of randomized clinical trials ( *n* is approximately 8,000) and systematic reviews ( *n* is approximately 50) conducted in populations with renal diseases. Your search identified a systematic review of statins in patients on dialysis [ 9]. This review included six randomized clinical trials, which were of short duration ( Table 1). Statins reduced total cholesterol (five studies, 357 patients; weighted mean difference, –53.74 mg/ dl [1.40 mmol/L]; 95% confidence interval [CI], –66.95––40.54) and LDL cholesterol levels (five studies, 357 patients; weighted mean difference, –55.40 mg/dl [1.44 mmol/L]; 95% CI, –69.90––40.90) similar to the general population, without any increase in the rate of adverse events (hepatotoxicity abnormal liver function test results or rhabdomyolysis-elevated creatine kinase levels). However, data on cardiovascular and cerebrovascular events in patients on dialysis were never looked at by the trials and, hence, not analyzed in this review. Since publication of that Cochrane review, which is currently being updated, a large statin trial in patients on dialysis (4D trial) came into the public domain. This trial found no significant difference in the rates of cardiovascular mortality and events in diabetic patients on dialysis receiving statins compared with those who did not receive statins.

**Table 1:**
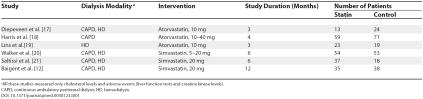
Characteristics of the Populations and Interventions in the Short-Term Randomized Controlled Trials of Statins Conducted in Patients on Dialysis Focusing on Surrogate End Points

In the Renal Health Library, there was no systematic review of randomized trials analyzing the role of statins in patients with earlier stages of chronic kidney disease (“predialysis” patients), and there was one non-Cochrane review that analyzed the role of statins in patients with kidney transplants [ 10]. This latter review concluded that in transplant recipients, statins may reduce clinical cardiac events, along with improvement in surrogate end points (lipid markers). In summary, these data mainly indicate that statins are effective in improving surrogate end points, but have never been strongly demonstrated to affect mortality and cardiovascular events. You therefore go further in your analysis of trials published after the systematic reviews, and, hence, not included in those analyses. In particular, you focus on individual clinical trials conducted in patients on dialysis, predialysis patients, or patients with renal transplants.

## Recent Clinical Trial Evidence for Use of Statins in Patients on Dialysis

Recently, the largest clinical trial of statins in patients on dialysis was published [ 11]. The 4D trial analyzed the efficacy of atorvastatin in 1,200 patients with diabetes on hemodialysis in Germany. The primary end point was a composite of death from cardiac causes, fatal stroke, nonfatal myocardial infarction, or nonfatal stroke—whichever occurred first. Even though the impact on cholesterol reduction was similar to that observed in the general population, atorvastatin had no statistically significant effect on the composite end point (relative risk [RR], 0.92; 95% CI, 0.77–1.10; *p* = 0.37). The use of atorvastatin decreased the number of cardiovascular events (fatal and nonfatal) significantly (RR, 0.82; 95% CI, 0.68–0.99; *p* = 0.03), but the impact on all-cause mortality was not significant in comparison with placebo (RR, 0.93; 95% CI, 0.79–1.08; *p* = 0.33).

The United Kingdom heart and renal protection (HARP) trial analyzed the safety and efficacy of simvastatin in three subsets of patients with chronic kidney disease, including predialysis, renal transplant, and also patients treated with dialysis [ 12]. It concluded that the use of simvastatin consistently decreased cholesterol levels in all subgroups, without additional toxicity (i.e., simvastatin therapy was not associated with excess risk for hepatotoxicity or rhabdomyolysis). Again, despite the relatively large sample size ( *n* = 448; patients on dialysis = 73), long-term outcomes, particularly mortality rates, were not reported in this trial.

## Recent Clinical Trial Evidence for Use of Statins in Predialysis Patients and Recipients of Renal Transplants

Tonelli et al. analyzed the role of pravastatin in 1,711 patients with creatinine clearance less than 75 ml/min and not on dialysis [ 13]. This study was a subanalysis of the larger cholesterol and recurrent events (CARE) trial that enrolled 4,159 patients with prior myocardial infarction and cholesterol levels greater than 240 mg/dl; it has been subsequently included in meta-analyses including the large Pravastatin Pooling Project and has been widely disseminated [ 14]. The study of Tonelli et al. concluded that the use of pravastatin decreased the incidence of major coronary events, with no impact on all-cause mortality ( Table 2). The study individually may not be adequately powered to detect the impact on all-cause mortality rates in predialysis patients with kidney disease, but the subsequent Pravastatin Pooling Project analyses, including additional information in predialysis patients from the West of Scotland coronary prevention study (WOSCOPS) and long-term intervention with pravastatin in ischemic disease (LIPID) studies, demonstrates a significant reduction in the risk of all-cause mortality with statins in these patients (RR, 0.81; 95% CI, 0.73–0.89; *p* = 0.03).

**Table 2:**
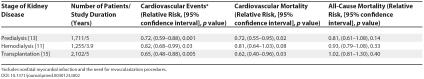
Results of the Three Major Statin Trials Reporting Patient-Level End Points in Patients with Chronic Kidney Disease

In the assessment of lescol in renal transplantation (ALERT) trial, the use of fluvastatin in patients with renal transplants has been found to decrease coronary events and cardiovascular mortality rates by an extent similar to that observed in patients without kidney disease, although there was no impact found on all-cause mortality [ 15]. In terms of cardiovascular end points, both the studies demonstrated that the benefits of statins in predialysis populations and in recipients of renal transplants are similar to those observed in the general population (patients without kidney disease) ( Table 2).

## Implications for Clinical Practice

Coming back to the original focus on our patient on dialysis, what does the current trial and systematic review evidence tells us about statins and cardiovascular disease in this scenario? The systematic reviews looked at surrogate end points alone. If these are validated surrogates, as in the general population, then statins reduce the levels of the surrogate and also the rate of the major event distal to the surrogate in the causal pathway of events. In summary, provided total cholesterol and LDL cholesterol levels are validated surrogate end points, statins reduce their levels, and this implies cardiovascular morbidity and mortality. The fact that the 4D trial found an effect on the surrogate but not on all-cause or cardiovascular mortality may indicate that initiation of statin therapy in patients on dialysis may be too late, and statin use should be considered in patients with chronic kidney disease before they reach dialysis. In fact, both predialysis patients and recipients of renal transplants significantly benefit from statins for both cardiovascular morbidity and mortality and all-cause mortality. In both patients in the predialysis stage and those with transplants, cardiovascular event and cardiac mortality rates decreased similarly to the general population (about 20%). This decrease was not found in patients on dialysis with diabetes, where much more severe comorbidities occur such that the benefits of statins are less likely to occur, in particular with respect to survival.

Hence, it is reasonable that despite absence of trial evidence in the dialysis population, statins should confer some benefit, and perhaps dose, duration, cointerventions, and other still unexplored aspects may play a role in determining whether they result in a survival advantage. Very likely, a meta-analysis including predialysis, dialysis, and transplant data will show a cumulative estimate of significant reduction for both cardiovascular and total mortality, and will demonstrate that the stage of chronic kidney disease (predialysis, dialysis, and transplant) is not an effect modifier. Such meta-analyses are being conducted. Furthermore, other randomized trials may address the question. These are also ongoing. In particular, there are two randomized trials currently in progress: the study of heart and renal protection (SHARP) trial, which will include 3,000 patients on dialysis and 6,000 predialysis patients, and the AURORA study, which will include 2,700 patients on hemodialysis [ 16]. Their results are awaited to build on current knowledge and to make a final informed decision on whether a statin is beneficial in dialysis, but there is insufficient evidence at present to say that statins should not be used, particularly in light of no substantial evidence of harm.

## What Will You Suggest to Your Patient?

Your patient comes for the scheduled follow-up visit. You tell him about the studies that looked at whether use of statins in his condition is supported by evidence from randomized trials. Statins have in fact been proven to decrease the cardiovascular event rates in patients with chronic kidney disease, and no substantial evidence of harm was demonstrated in these patients. In comparison with trials conducted in all other populations, there are no major counterarguments that statins should be harmful to him, although the only trial conducted in individuals with diabetes on dialysis did not confirm any cardiovascular or survival benefit—perhaps for methodological reasons, mainly including sample size/low power—selecting a high-risk population where major benefits were not likely to be seen, even though harm was not observed. Despite your strictly “evidence-based” approach to decision making, you decide with your patient that you will follow the current recommendations for statin use by the National Kidney Foundation–Kidney Disease Outcomes Quality Initiative guidelines, and look forward to the results of currently ongoing trials. In fact, you discuss with your patient that you will seek information about whether he himself may be enrolled in one of the ongoing trials of statins.

## Abbreviations

CI: confidence interval
GFR: glomerular filtration rate
HDL: high-density lipoprotein
LDL: low-density lipoprotein
RR: relative risk
